# Exploring Relative Age Effects in Youth Ice Hockey Through a Single Team Case Study and Composite Narratives

**DOI:** 10.3389/fspor.2021.658953

**Published:** 2021-05-19

**Authors:** David J. Hancock

**Affiliations:** Memorial University of Newfoundland, St. John's, NL, Canada

**Keywords:** birthdate, participation, qualitative analysis, case study design, relative age effects, youth sport

## Abstract

Relative age effects (when birthdate influences participation or performance) in sport potentially influence the experiences of sport participants, including athletes, parents, and coaches. Nearly all existing literature on relative age effects adopts a quantitative approach, limiting our understanding of the phenomenon. Thus, the purpose of this unique study was to explore relative age effects using an instrumental, descriptive case study approach with one elite, youth, male ice hockey team. This context was chosen given the high prevalence of relative age effects among such groups. Participants included 20 athletes, 19 parents, and two coaches from one team. Data were collected through biometric measures, semistructured interviews, and participant observations. Results were presented as five composite narratives: relatively older athletes, relatively younger athletes, parents of relatively older athletes, parents of relatively younger athletes, and coaching staff. The narratives demonstrated unique relative age experiences for each group of participants. Discussion aligns the results with the social agents model that explains relative age effects. Practical recommendations for sport participants are also provided.

## Introduction

There are a number of factors that influence the acquisition of expertise in any field. In sport, Baker and Horton (2004) expressed the importance of considering primary and secondary influences on athletic expertise. Primary influences such as genetics and training directly affect expertise, whereas secondary influences such as sport maturity and depth of competition indirectly affect expertise. Despite the term, researchers generally describe secondary influences as equally important as primary ones and have spent considerable efforts understanding secondary influences that affect expert development. Baker and Horton outlined several factors that indirectly influence talent development including cultural importance of sport (e.g., Robinson, 1998), access to quality coaching (e.g., Côté and Gilbert, 2009), and familial support (e.g., Côté, 1999). Secondary influences extend to birth advantages, such as birthplace effects (Côté et al., 2006) and the focus of this study, relative age effects (Cobley et al., 2009).

“Relative age effects” occur when one's birthdate influences sport participation or performance (Musch and Grondin, 2001). This phenomenon is prevalent in youth sport, where children are frequently grouped into 1-year age cohorts. In most ice hockey organizations, for instance, children compete with peers born in the same calendar year (e.g., Barnsley and Thompson, 1988). Thus, a child born January 1 is 364 days older than a child born December 31, yet the two children compete in the same age division. Relatively older athletes are afforded participation (i.e., increased enrollment; Turnnidge et al., 2014) and performance advantages (i.e., increased representation on elite teams; Hancock et al., 2013b), while relatively younger athletes are more apt to drop out (Delorme et al., 2010). Relative age effects exist in several sports including ice hockey (e.g., Barnsley et al., 1985; Nolan and Howell, 2010), association football (e.g., Cobley et al., 2008; Figueiredo et al., 2019), rugby (e.g., Lemez et al., 2016; Cobley and Till, 2017), tennis (e.g., Ulbricht et al., 2015; Gerdin et al., 2018), skiing (e.g., Baker et al., 2014; Müller et al., 2015), basketball (e.g., Delorme et al., 2011; Arrieta et al., 2015), and gymnastics (e.g., Baker et al., 2014; Hancock et al., 2015). Furthermore, relative age effects exist in Australia (e.g., van den Honert, 2012), Brazil (e.g., Okazaki et al., 2011), Canada (e.g., Nolan and Howell, 2010), France (e.g., Delorme et al., 2011), Germany (e.g., Ulbricht et al., 2015), UK (e.g., Till et al., 2014), and the USA (e.g., Giacomini, 1999), to name a few.

Relative age effects facilitate sport inequities that are driven by arbitrary dates^1^. These inequities potentially lead to long-term consequences that ought to be concerning to sport organizations and governments. Specifically, one possible consequence is a reduced talent pool at older age categories, as relatively younger athletes are more likely to discontinue sport before attaining their potential. Relative age effects ought to be concerning as well from the athlete's perspective. A possible consequence of a system that advantages relatively older athletes is health concerns, as relatively younger athletes miss out on important physical activity and social opportunities when they drop out of sport. For both reasons, it is imperative to gain a deeper understanding of relative age effects.

Recently, researchers began conceptualizing models to explain relative age effects. The model most relevant to this study is the social agents model by Hancock et al. (2013a). The authors described relative age effects as a social phenomenon, heavily influenced by parents', coaches', and athletes' actions and perceptions. Hancock et al. (2013a) proposed that parents register relatively older children in sport at younger ages, and these initial advantages lead to further advantages known as a Matthew effect (Merton, 1968). This initial enrollment bias affords relatively older children more sport experience, training, instruction, and skill development than their relatively younger counterparts, leading to potential performance advantages in subsequent years. The authors also suggested that parents have higher sport expectations for their relatively older children, leading to a Pygmalion effect (Rosenthal and Jacobson, 1968). For instance, a parent's heightened expectation for athletic success could potentially transform behavior whereby they register their child for additional sport clinics. In turn, this leads to a behavioral change within their child, whereby they believe they are talented and commit to additional practice time. In such scenarios, athletes confirm their parents' expectations, completing a self-fulfilling prophecy. According to the model (Hancock et al., 2013a), coaches are provided with a relatively older talent pool due to the initial enrollment bias. The authors also hypothesized that, similar to parents, coaches form high expectations of relatively older athletes, spurring another self-fulfilling prophecy or Pygmalion effect (Rosenthal and Jacobson, 1968). An example is coaches providing more instruction to relatively older athletes, who improve their skills beyond that of relatively younger athletes. Finally, based on parents' and coaches' expectations, athletes might then form new self-expectations regarding their ability and relative age, known as a Galatea effect (Merton, 1948), which influences relative age effects.

The model by Hancock et al. (2013a) places social agents at the root of relative age effects. Essentially, if parents and coaches had equal expectations and perceptions for relatively older and younger athletes, then relative age effects would dissipate, if not disappear. The goal of this paper is not to debate if this is true; however, the authors' claims are substantiated in that social agents clearly influence relative age effects. This warrants further investigation into social agents and relative age effects.

With few exceptions (Chittle, 2020; Lidor et al., 2021), the research on relative age effects has been acquired through quantitative approaches, often relying on archival methods. It is vital that researchers interact directly with social agents to understand relative age effects. Thus, the purpose of the present study was to use a case study approach to investigate how athletes, parents, and coaches experience relative age effects. This includes their awareness of the effect, its existence within the team, and their perceptions of the effect on athletic development. The study design immersed the researcher in one ice hockey team's culture, providing a deeper understanding of the influence of relative age effects on the team.

## Materials and Methods

The procedures described herein were approved by the author's Institutional Review Board. All participants provided informed consent/assent prior to participation.

### Researcher Paradigm

The researcher aligns with the pragmatic paradigm (Dewey, 1931; Morgan, 2014), whereby he is not concerned with debates of truth and reality, since such debates cannot be conclusively ruled upon (Giacobbi et al., 2005). The pragmatic paradigm is effective in eliciting an understanding of “real-world” problems and focuses on finding meaning that is useful to the studied population (James, 1907; Rorty, 1990; Maxcy, 2003). Given that relative age effects have a tangible influence on youth sport participants' experiences, it seemed appropriate to adopt the pragmatic paradigm. Guided by this paradigm, all methodological decisions were based on increasing the relevance of this research to youth sport participants (e.g., athletes, parents, and coaches). This was the impetus for a case study design. Rather than sample a number of sports or teams, the researcher studied one team to capture participants' experiences with relative age effects and provide a depth of understanding that is lacking when athletes are studied using large, cross-sectional, quantitative designs.

### Case Study Design

The study purpose led the researcher to conclude that a case study design would be best suited to (1) uncover meaningful results by including multiple sport participants (i.e., athletes, parents, and coaches) and (2) frame the results to be useful and tangible for the ice hockey community. Specifically, the researcher employed an instrumental (using one case to understand other cases; Stake, 1995), descriptive (describing a phenomenon in its real-world context; Yin, 2014) case study approach with one ice hockey team to understand participants' experiences related to relative age effects in the youth sport environment.

### Participants

The strongest relative age effects are typically found in male, physically demanding, team-oriented, competitive, and regionally popular sports—especially when athletes are in or nearing puberty (Cobley et al., 2009; Hancock, 2020). The chosen team, therefore, was a North American, “AAA” (highest competitive level for youth), male ice hockey team. Athletes were 14 or 15 years old but born during the same calendar year (i.e., some had their 15th birthday before the season ended). The sample included all 20 athletes, 19 parents, one head coach, and one assistant coach.

### Data Collection

To build a strong case study, data collection ought to arise from multiple sources (Yin, 2014). Herein, data collection included three sources as per Yin's (2014) categorization of case study evidence: direct observations, semistructured interviews, and participant observations.

#### Direct Observations

The researcher selected three measures for direct observations. First, athletes self-reported their birthdates. North-American ice hockey establishes age divisions based on the calendar year, with the relatively oldest athletes born in January. Athletes were considered relatively older if they were born between January 1 and June 30, while relatively younger athletes were born from July 1 to December 31. This yielded 13 relatively older athletes and seven relatively younger athletes. As the parents of one athlete did not consent to participate, there were 12 parents of relatively older athletes and seven parents of relatively younger athletes.

Second, relative age effects are often—and perhaps falsely—attributed to the physical differences that exist between athletes of differing ages (Hancock, 2020). It was important, therefore, to collect athletes' basic biometric measures to provide context regarding how athletes of differing relative age might experience physical differences. The researcher measured height, weight, and strength, since sport scientists often cite these as advantageous for relatively older athletes (Till et al., 2014). For height, a tape measure was secured to a wall and athletes were measured without wearing shoes or hats. Athletes were weighed on an electronic scale while wearing only their moisture-wicking base layer that is traditionally worn under ice hockey equipment. Next, athletes performed a grip-strength test, following the procedures outlined by the American College of Sports Medicine (2009).

For the third direct observation, participants estimated biometric rankings, providing insights into their awareness and perceptions of the relationship between relative age and growth. Athletes ranked themselves compared with their teammates for age, height, weight, and grip strength. As an example, athletes were asked, “With #1 being tallest and #20 being shortest, where do you rank your height compared to your teammates?” Similarly, parents ranked their child compared with his teammates for each biometric measure. The head coach and assistant coach rank ordered every athlete on each of the biometric measures.

#### Semistructured Interviews

The researcher began by using a random number generator to select 10 athletes (which yielded six relatively older and four relatively younger athletes) to engage semistructured interviews. The social agents model (Hancock et al., 2013a) that guided this research suggests parents' behaviors influence athletes' expectations. Thus, to see if similarities existed between parents' and athletes' responses, the parents of the 10 interviewed athletes also engaged in semistructured interviews. Parents could interview together (*n* = 2) or have one person represent the parent unit (*n* = 8). When two parents were interviewed together, they were treated as one participant. Lastly, the head coach was interviewed after the athlete and parent interviews. Upon completing these interviews, it was evident that data saturation was reached; thus, no further interviews were conducted.

Interview questions were guided by the study purpose and aligned with the instrumental, descriptive case study design (i.e., understanding the relative age effect through the lens of one youth ice hockey team). Questions were shaped by a pragmatic paradigm, with the goal of discovering information that was useful to youth ice hockey participants. Interviews began by identifying participants' knowledge of relative age effects (e.g., What, if anything, can you tell me about relative age effects?). Next, participants were asked about perceptions of relative age effects (e.g., Do you have any guesses as to why relatively older athletes are selected to elite teams?). Third, participants were asked about the link between relative age and size/ability (e.g., Do you think birthdate can influence someone's chances of being a good ice hockey player?). Finally, participants were asked personally relevant questions (e.g., If given the chance, would you change your [son's] birthdate?). The coach was asked if he made team selection decisions based on birthdate, height, weight, and/or strength. Probes were placed in the interview guide to elicit more information from participants. After developing the initial interview guide, the researcher conducted a pilot interview with a colleague who was well-versed in qualitative methods and relative age effects. Based on his feedback, the researcher made minor changes to question wording. Interviews took place after the season in the participants' homes, with athletes interviewed before their parents. The researcher did not provide participants with any information about relative age effects before the semistructured interviews. The average and anticipated interview lengths were as follows: athletes (10:51 min; 15 min), parents (18:24 min; 20 min), and head coach (32:09 min; 30 min). Interviews were transcribed verbatim by the researcher for later data analysis.

#### Participant Observations

According to Yin (2014), participant observations involve the researcher actively engaging with the participants, rather than being a passive observer. In this study, the researcher interacted with the participants on several occasions for the purposes of recruitment, informed consent/assent, and collecting biometric measures. These interactions spanned several weeks whereby the researcher attended six practices and two games (a total of 20 h). It was not necessary for the researcher to remain at the practices and games for their entire duration, but in doing so, he was able to develop rapport with participants, observe their behaviors, and engage in conversations with participants (primarily parents, but also athletes and coaches to a lesser extent). After each practice/game, the researcher made field notes about his observations that helped to inform probing questions during the interviews, interpret the interviews, and provide context to the composite narratives.

### Data Analysis: Composite Narratives

Composite narratives (Willis, 2019) allow researchers to reflect on participant data and communicate a story to the reader that captures participants' emotional truth (Orbach, 2000; Wertz et al., 2011). Several factors led the researcher to choose composite narratives. First, conducting inferential analyses did not align with the case study design—especially given that quantitative measures were taken from only 20 athletes. Second, throughout data collection, it was evident that each participant group (i.e., athletes, parents, and coaches) had unique experiences with relative age effects. Third, composite narratives offer non-academic audiences a better understanding of phenomenon than traditional qualitative methods that employ codes and themes to communicate findings (Willis, 2019), which is a crucial tenet of the researcher's pragmatic paradigm. All sources of data collection were synthesized into five composite narratives representing (1) relatively older athletes, (2) relatively younger athletes, (3) parents of relatively older athletes, (4) parents of relatively younger athletes, and (5) the coaching staff.

Quality composite narratives must be created transparently (Willis, 2019). Herein, the researcher incorporated five main principles. First, the researcher played competitive ice hockey for 13 years, has refereed competitive ice hockey for 23 years, and has studied relative age effects for 13 years. This led participants to treat him as an “insider” with excellent ice hockey acumen. Second, multiple sources of information informed the composite narratives. Whereas, the interviews accounted for most of the content, each athlete narrative (i.e., relatively older and younger) included the group's average birthdate, height, weight, and strength. Additionally, participants' estimated rankings and participant observations provided context to the narratives that helped understand how relative age influenced the respective groups. Third, presentation of the narratives was carefully chosen. Each narrative was assigned a pseudonym. Though differing opinions exist (e.g., Wertz et al., 2011; Willis, 2019), the composite narratives were written in third person to highlight the participant-researcher interactions. Any direct quotes in the composite narratives were gleaned from the interviews verbatim. Fourth, choices regarding the content to include in the composite narratives were important. The researcher relied on his background and experiences in ice hockey and relative age effects to select content he deemed most relevant (i.e., directly or tangentially related to relative age effects) to the respective composite narratives. Fifth, the composite narratives represent the researcher's interpretation of participants' data. After presenting the composite narratives, the researcher then unpacked their meaning in the discussion.

### Methodological Quality and Rigor

The researcher enacted three techniques to enhance quality and rigor. First, the study maintained methodological coherence (Mayan, 2009), as the decisions in the study (e.g., purpose, case study design, composite narratives, and discussion) all aligned with the researcher's pragmatic paradigm. Second, to ensure a rich description (Creswell, 1998) in the composite narratives, the researcher had extended contact with the participants over the span of six weeks, which included attending six practices and two games (a total of 20 h). Third, Yin (2014) outlined three criteria to assess the quality of descriptive case study designs: construct validity, external validity, and reliability. Construct validity refers to providing operational definitions related to the research topic and design (Yin, 2014). The researcher established construct validity by clearly defining relative age effects and past findings in the literature review, including multiple sources of data collection, and ensuring participants understood relative age effects during the interviews. External validity entails developing a research question that addresses how something happens (Yin, 2014). For external validity, the data collection was directed by a “how” research question, namely, “How do athletes, parents, and coaches experience relative age effects?” External validity was further enhanced by the inclusion of the social agents model (Hancock et al., 2013a). Thus, there is potential for the results herein to be generalized to similar sport contexts where relative age effects might be influenced by social agents. Reliability relates to the ability of other researchers to follow the same procedures and reproduce similar results (Yin, 2014). Through precise description of the case study methods and composite narrative decisions, the study attained reliability.

## Results

### Derek (Relatively Older Athlete)

Born March 22, Derek is among the older athletes on the team, having already turned 15 years old before the season ends. He has played ice hockey for 10 years and now plays defense. Derek seems excited about the biometric measurements. Derek stands 173 cm tall (9th tallest), weighs 68 kg (9th heaviest), and has a grip strength score of 37 kg (10th strongest). Compared with his teammates, Derek predicts he is 9th tallest, 8th heaviest, and 8th strongest. Before the study, Derek had never heard of relative age effects, but now explains the effect as, “It has something to do with your birthday.” Derek thinks being relatively older leads to physical advantages, “They tend to grow up faster. They tend to get bigger before everybody else. So it gives them a performance edge, I suppose.” However, he indicates that relative age is not the sole factor for performance, “I just think it all depends on how bad you want it, how hard you work, you know? I don't think [your age] really matters.” When asked if athletes are selected to teams simply because of size, Derek equivocates, stating it is easier for bigger athletes to get noticed, but stops short of saying it is a conscious process. Probed about whether a poor-skating, 200-cm-tall athlete would get selected to a team, he relents, “Yeah, because you can improve skating.” Oppositely, Derek recounts a situation where a smaller athlete got deselected because of his size, “He didn't get chosen…and he was better than [the athlete who was selected]; he didn't make it just ‘cause he was, like, half the size.” Though Derek believes one's birthdate might influence chances of becoming a good ice hockey player (“Yeah, probably, because they may get introduced to the sport earlier and they may develop their skills earlier on, so they're better at it.”), he emphatically disagrees that his birthdate influences his height, weight, or strength and is not certain it has influenced his chances of becoming a good ice hockey player.

### Colin (Relatively Younger Athlete)

Born September 24, Colin is relatively younger compared with his teammates and will not turn 15 years old until next season. He plays offense, accumulating 7 years of ice hockey experience, though several years were in non-competitive divisions. Colin is noticeably shorter and lighter than Derek, and he is unenthusiastic about the biometric measures. The measures confirm his biometric disadvantages. Colin stands 167 cm tall (13th tallest), weighs 59 kg (13th heaviest), and scores 34 kg (12th strongest) on the grip strength test. Colin holds a favorable perception of his biometrics, estimating he is 12th tallest, 11th heaviest, and 10th strongest on the team. He had never heard of relative age effects before the study but now has a better grasp of the effect, “It is like if you are born between certain months, you're supposed to be better at hockey or better at sports.” When probed, Colin homes in on opportunities such as entry age, experience, and practice to explain why relatively older athletes have advantages, “Maybe just because they're a little bit older, so they'd have more time practicing.” Colin has witnessed situations where athletes were selected to teams simply because of their size, “Quite often, because [coaches] just see a big kid and just love them. I didn't like that very much…[be]cause obviously I'd choose the person that's better—not because of their height or weight, but because they're better.” Similarly, he experienced situations where athletes were deselected from teams based on size, “One of my friends got cut from a team. The coach told him he was too small to be on the team…he wasn't on the team because he was too small.” Colin appeared receptive to the idea that birthdate might influence chances of becoming a good ice hockey player (e.g., “I think it could, but at the same time it might depend on their work ethic too.”) but disagreed that his birthdate influenced his height, weight, strength, or chances of becoming a good ice hockey player.

### Lesley (Parent of Relatively Older Athlete)

Lesley is Derek's mother, who is one of the oldest athletes on the team. She has not heard of the relative age effect before beginning the study but seems curious about how it might have influenced Derek. Lesley holds a favorable view of Derek's biometrics, predicting he is 8th tallest, 8th heaviest, and 7th strongest (he was 9th, 9th, and 10th, respectively). Since the study began, Lesley has spoken with other team parents about relative age effects and realizes that birthdate might influence ice hockey success, “I've wondered if some of those kids that are at the end of the birth year, if they tend not to go as far because they are smaller.” As Lesley discusses her son, it is evident she sees a link between birthdate and physical prowess, “The older [athletes] should be typically the larger or the stronger or grow faster—you know, they're more mature. They're ahead of the others with the time they've had, right?” Despite this, Lesley indicates athletes were rarely chosen to elite teams because of their size, and further states that Derek's size is more a genetic factor than related to his birthdate, “I don't see how the birthdate really—I mean he's going to grow at the speed of his genetics… I don't think it matters what month you're born when you're going to hit puberty.” Lesley shares Derek's belief that birthdate could influence success in ice hockey (“The January, February, March birthdays probably being the better hockey players or having the chance to be the better hockey players—because of being older, being more mature, hitting the maturity quicker than the younger kids. I don't know if that's 100% true, but I would say that it's a probability that there's more with earlier birthdates in the year.”) but does not believe this has been the case for Derek. Lastly, Lesley states that Derek's birthdate never influenced sport enrollment decisions, “No, never.”

### Andrew (Parent of Relatively Younger Athlete)

Andrew is Colin's father, who is one of the youngest athletes on the team. He has heard of relative age effects and is intrigued about what the study might reveal. Andrew overestimates Colin's biometrics, predicting he is 11th tallest, 12th heaviest, and 8th strongest (he was 13th, 13th, and 12th, respectively). Andrew heard about relative age effects from the book *Outliers*, as well as a conversation with his daughter's soccer coach. He says his son's later birthdate piqued his interest in relative age effects. Asked why relatively older athletes have sport advantages, Andrew focuses on physical differences at young ages, “There's a significant difference between a 10-year-old born in January than a 9-year-old born in December. And so it may be early on because of those differences, those kids get frustrated or whatever and find something else to do.” Continuing, Andrew highlights that relatively older athletes likely draw coaches' attention at tryouts because of their size and that many coaches select athletes based on physical characteristics rather than skill, “I do truly believe—particularly at this age and even last year or maybe the year before that—I think coaches specifically chose kids based on their size at a tryout.” When speaking of birthdate and size, Andrew says, “It's probably more genetics than anything else. I was the same as [Colin]—birthdate doesn't make a big deal.” Andrew is uncertain if there is a link between birthdate and success in ice hockey, “I mean, it might. I just think there's so many other determining factors…it might be one factor out of 10 or 15 other ones. But I wouldn't put it at the top. There's a lot of other factors that I would put ahead of birth[date].” Referring to Colin's birthdate influencing his chances at being a good ice hockey player, Andrew states “I don't know, but I don't think [coaches are] looking at the birthdates. To the best of my ability I think they're more looking at the height, weight over birthdate.” Lastly, Andrew has never made sport enrollment decisions based on Colin's birthdate.

### Paul (Coaching Staff)

Paul enthusiastically agrees for his team to be in the study, saying he is aware of relative age effects and would like to contribute to the growing body of knowledge. Paul finds the estimated rankings challenging since he does not focus on biometrics. Examining absolute differences between actual and estimated rankings (e.g., if the 10th tallest athlete was estimated as 8th or 12th tallest, the difference was “2”), Paul is more accurate with visible metrics (height = 1.1 average difference; weight = 3.5) than non-visible metrics (age = 4.8; strength = 6.4). In the interview, it is evident that Paul knows more about relative age effects than any of the athletes or parents in the study. He thoroughly describes the effect, including the futility of changing selection dates (aligned with Helsen et al., 2000). Paul explains relatively older athletes' sport advantages, “11 months is a long time when you're a 5- or 6- or 7-year-old. The competitive advantages that would go with being born in January of a year vs. December and those kids competing against each other would be the same as a baby learning to crawl at a certain time vs. a baby that's born later and comparing those two babies in terms of their motor development.” Whereas Paul does not select athletes solely due to their size, he concedes it influences selection decisions when comparing similarly skilled athletes, “If I have two athletes of equal talent, I'll go with the bigger athlete. They are more durable, typically. They bring a physical component beyond the skill set. If they're bigger, they command more respect on the ice.” Paul also believes relatively older athletes have better odds of excelling in ice hockey due to their size, “So you see him out there and now he's got 5 inches or 6 inches on some of these kids and probably 35, 40 pounds on some of them. So yeah, I think it does [increase success] because even if he's not the most talented kid out there, he makes room for himself just from other kids getting out of the way.” Ultimately, Paul considers size and strength during team selections and acknowledges these characteristics are likely related to relative age.

## Discussion

The researcher used an instrumental, descriptive case study design with one elite, male, youth ice hockey team to understand participants' experiences with relative age effects. Five composite narratives with multiple sources of data represented relatively older athletes, relatively younger athletes, parents of relatively older athletes, parents of relatively younger athletes, and the coaching staff. Since the interpretation of the composite narratives aligns with the social agents model for relative age effects, this section follows the same order Hancock et al. (2013a) outlined in their article (i.e., parents, coaches, and athletes).

### Parents

Lesley (parent of a relatively older athlete) and Andrew (parent of a relatively younger athlete) shared some similar relative age experiences. For instance, both parents acknowledged relatively older athletes might have size advantages over relatively younger athletes. Lesley and Andrew believed this could manifest into ice hockey success through better skills, but more so, they believed increased size could result in being selected to more elite teams, resulting in experiential and practice advantages. Despite these statements, both parents stated their child's growth was primarily driven by genetics and not birthdate. In this sense, they did not believe their child's size (dis)advantages were due to relative age.

Andrew also expressed some experiences that were markedly different than Lesley. Specifically, Andrew believed that team selection decisions were often based on size and not skill. It is not surprising that Andrew reported this experience, given that his son was noticeably smaller than other players and team selection decisions potentially negatively influence his son. In other words, Andrew is more cognizant of size disadvantages due to their potential negative consequences—this contrasts Lesley's experience, who would have no reason to be preoccupied with size during team selection decisions. Since Andrew's son overcame his size disadvantage to be selected to an elite team, it might explain his more favorable biometric rankings, as he views his son has proven himself to be similar to other players on the team.

The results do not support that parents hold different perceptions of relatively older and younger athletes that would explain Matthew effects (Merton, 1968). It is important to note, however, that parents were interviewed 10 years after they could have registered their children for ice hockey, perhaps preventing accurate recall of those decisions. Secondly, this study did not include parents of athletes who played in non-competitive ice hockey or parents of athletes who dropped out of ice hockey—their enrollment decisions might be very different than Lesley's and Andrew's. For Pygmalion effects (Rosenthal and Jacobson, 1968), both parents have expectations and perceptions regarding relative age effects that have the potential to influence their sons. Lesley perceives ice hockey success to be about growth and maturation, Derek's advanced size is related to genetics, and team selections are rarely based on size. These perceptions might lead Derek to feel more secure about his odds of becoming a good ice hockey player as these factors are advantageous for him. Meanwhile, Andrew perceives ice hockey success to be about many factors, Colin is small because of genetics, and team selections are frequently based on size. This could lead Colin to believe he has too many negative factors to overcome as he pursues ice hockey success, potentially leading to drop out. The researcher believes both parents' perceptions of (dis)advantages are based on relative age (even if the parents did not use the term) and demonstrate potential Pygmalion effects (Rosenthal and Jacobson, 1968).

### Coaching Staff

Paul had significant knowledge about relative age effects. He explained the effects in terms of development, maturity, and experiential advantages, leading him to conclude that shifting selection dates would simply shift who is advantaged by the system. Paul claimed he did not focus on his players' relative age, height, weight, and strength, which was supported by how challenging he found the estimated biometric rankings. However, he also stated that if he had to choose between two similarly skilled athletes for his team, he would choose the bigger player with the assumption that player is more durable and creates more space on the ice.

Paul's insights very much align with the social agents model (Hancock et al., 2013a). First, his description of an 11-month age difference at young ages is nearly identical to Hancock et al. (2013a) explanation of how Matthew effects (Merton, 1968) benefit relatively older children (i.e., early age advantages lead to more experience, practice, instruction, and, therefore, team selections). Second, when faced with choosing one athlete out of two similarly skilled athletes, Paul selects the bigger player because he perceives many positive benefits from this athlete (e.g., durability, ability to create space, can intimidate opponents, etc.). This is, in fact, the beginning of the Pygmalion effect (Rosenthal and Jacobson, 1968), which is potentially grounded in false beliefs. To the researcher's knowledge, there is no relationship between a youth athlete's size and their durability, fearlessness/courage, or ability to intimidate opponents. Nevertheless, Paul holds this belief to be true, which influences his team selection decisions. This likely benefits relatively older athletes who get more practice, playing time, and instruction, thereby leading to performance improvements and completing Paul's self-fulfilling prophecy (Merton, 1948).

### Athletes

The athlete interviews were shorter than expected, as many interviewees provided quick responses even after probing from the researcher. Nevertheless, as a collective group, their responses provide interesting insights into relative age effects. Derek's relative age experiences exemplified a stereotypical relatively older athlete. He was taller, heavier, and stronger than Colin and held a favorable perception of his biometric rankings. He entered the study without having heard about relative age effects and ended the study with little improvement of that knowledge. Initially resistant to the idea that size was a determining factor in team selection decisions, Derek eventually conceded that it is a factor for athletes at the extreme ends of the spectrum—though he was clear to point out that birthdate and size had not influenced his success in ice hockey. Reflecting on Derek's experiences, his approach mirrored that of someone with privilege in that he did not see a connection between his relative age and size, did not care to learn much about relative age effects, and did not think size was much of an advantage in ice hockey until the researcher probed more on the issue.

Oppositely, Colin experienced what would be expected of a relatively younger athlete. He was shorter, lighter, and weaker than Derek but ranked his biometrics better than reality. By the end of the study, Colin demonstrated superior relative age effects knowledge, possibly because it held stronger meaning for him. Colin's father indicated he became interested in relative age effects given his son's later birthdate—perhaps Colin's curiosity was sparked in the same way. Additionally, Colin was convinced that size is an instrumental factor in team selections and provided several examples of this. Again, his experiences of being relatively younger and smaller might have contributed to stronger perceptions of size and team selection decisions. Despite these experiences, Colin did not believe that his birthdate or size influenced his ability to succeed in ice hockey. While it is encouraging that Colin's experiences did not negatively affect his view on ice hockey success, it is important to acknowledge that his experiences are that of an athlete who was selected to the most elite ice hockey team in his region, which might differ greatly from an athlete who was deselected from the same team or who played non-competitive ice hockey.

According to the social agents model (Hancock et al., 2013a), Galatea effects (Merton, 1948) occur when external expectations and perceptions are placed on athletes, thereby influencing their expectations, perceptions, and behaviors. In this case study, it appears that Derek's and Colin's experiences and perceptions of relative age effects align with their parents. Derek and his mother both shared the belief that birthdate and size might influence ice hockey success but did not believe it was a significant factor nor that it was the case for Derek. Colin and his father, on the other hand, were both more mindful of relative age effects. They expressed more relative age effects knowledge, stated birthdate could contribute to ice hockey success, and believed size was important to team selection decisions (though again, did not believe Colin's odds for future success would be predicated on relative age). It would seem, then, that Derek and Colin have formed perceptions about relative age effects that align with external expectations and perceptions from their parents—a Galatea Effect (Merton, 1948).

### Sport Participants

A main tenet of the pragmatic paradigm is ensuring the data are useful for the studied population. The following list provides six evidence-based recommendations taken from this study that are useful for sport participants.

1. Sport governing bodies should provide coaches with information on relative age effects and guidance on how to avoid relative age biases during team selections (i.e., setting objective selection criteria before tryouts to direct coaches' attention to athletes' skills rather than size).
2. Coaches ought to critically reflect on how relative age effects, or related factors such as size, might influence their team selection decisions.
3. Parents and coaches should learn more about relative age effects including the importance of having high expectations of all athletes regardless of relative age (i.e., expect all athletes to reach their potential and, for youth, do not place limits on what their potential could be).
4. Coaches can ensure that athletes understand what controllable factors led to them to being selected to, or deselected from, the team.
5. Sport governing bodies could provide athletes and parents with information to help them understand talent development and primary/secondary influences on expertise.
6. Sport scientists should work with sport governing bodies to explore why relatively younger athletes have lower initial enrollment rates.

### Conclusion

This research contributes to the existing literature by demonstrating different relative age experiences for relatively older athletes, relatively younger athletes, parents of relatively older athletes, parents of relatively younger athletes, and coaches, all of whom were involved with one elite, youth, male ice hockey team. This is an important realization in relative age effects research and highlights the experiences of individual athletes, as opposed to identifying large cohort trends. Though the unique case study design has limitations (e.g., small sample size and challenges with generalizability beyond the studied context), it is evident that qualitative designs are beneficial to understanding relative age effects. Future researchers ought to consider more theory-based studies on relative age effects, such as exploring parents' roles in Matthew effects (Merton, 1968), investigating Pygmalion effects (Rosenthal and Jacobson, 1968) after team selections, and understanding how parents' and coaches' perceptions might initiate Galatea effects (Merton, 1948). Ultimately, such research will broaden our understanding of relative age effects in sport.

## Data Availability Statement

The raw data supporting the conclusions of this article will be made available by the author, without undue reservation.

## Ethics Statement

The studies involving human participants were reviewed and approved by Institutional Review Board; Indiana University. Written informed consent to participate in this study was provided by the participants' legal guardian/next of kin.

## Author Contributions

DH completed all phases of the research.

## Conflict of Interest

The author declares that the research was conducted in the absence of any commercial or financial relationships that could be construed as a potential conflict of interest.

## Footnotes

^1^Changing selection dates is ineffective, as advantages shift along with the selection dates (see Helsen et al., 2000).
